# Nonadiabatic Charge Carrier Dynamics in Rh-Doped BaTiO_3_ for Photocatalytic Water Splitting

**DOI:** 10.3390/molecules31142497

**Published:** 2026-07-17

**Authors:** Talgat M. Inerbaev, Fatima U. Abuova, Aidana G. Balabay, Aisulu U. Abuova, Dmitri S. Kilin

**Affiliations:** 1 Institute of Physical and Technical Sciences, L. N. Gumilyov Eurasian National University, Astana 010000, Kazakhstanabuova_fu@enu.kz (F.U.A.);; 2 Vernadsky Institute of Geochemistry and Analytical Chemistry, Russian Academy of Science, 119991 Moscow, Russia; 3 Department of Chemistry and Biochemistry, North Dakota State University, Fargo, ND 58108, USA

**Keywords:** photocatalytic water splitting, barium titanate, charge carrier recombination, reduced density matrix, Redfield theory

## Abstract

In this work, we performed a comprehensive first-principles investigation of the electronic structure, charge carrier relaxation dynamics, and photoluminescence properties of Rh-doped BaTiO_3_, with a focus on photocatalytic water splitting applications. By combining hybrid DFT (HSE06), DFT+U, and Redfield theory, we elucidated how the doping site (Ti vs. Ba), dimensionality (bulk vs. surface), and aqueous environment govern the nonequilibrium behavior of photogenerated electron–hole pairs. Rh occupying Ti sites on the (001) surface exhibits a unique combination of extended visible-light absorption, ultrafast non-radiative relaxation, and efficient charge separation. These characteristics establish it as a promising photoanode material for photoelectrochemical water splitting.

## 1. Introduction

The growing global demand for environmentally friendly and cost-effective energy sources has stimulated extensive research into renewable energy technologies. Among these, photoelectrochemical (PEC) water splitting has emerged as a promising approach for hydrogen production due to its affordability and environmental compatibility. Since the pioneering work of Honda and Fujishima in 1972, who first demonstrated hydrogen production using TiO_2_ photoelectrodes [1], significant progress has been made in developing materials and systems for solar-driven water splitting. In a typical PEC configuration, sunlight is absorbed by semiconductor materials to generate charge carriers that drive the hydrogen evolution reaction and oxygen evolution reaction [2,3].

The efficiency of PEC water splitting depends critically on the properties of the semiconductive electrodes. Ideal materials should possess a low band gap for visible light absorption, suitable band edge positions for water redox reactions, high stability, and low cost [4,5]. Transition metal oxides, particularly titanates, have attracted considerable attention due to their stability and suitable band structures [6]. However, their wide band gaps (e.g., 3.2 eV for BaTiO_3_) limit light absorption primarily to the ultraviolet region, which constitutes only a small fraction of the solar spectrum [7]. Consequently, extensive efforts have been directed toward band gap engineering of these materials to extend their optical response into the visible range.

Among the various strategies explored, doping with transition metals has proven effective in modifying the electronic structure of perovskite oxides. In particular, rhodium (Rh) doping has been shown to introduce mid-gap states that enable visible light absorption in SrTiO_3_ and TiO_2_ systems [8,9,10]. Rh-doped SrTiO_3_, for instance, exhibits remarkable photocatalytic activity for H_2_ evolution under visible light [11,12,13], although it fails to oxidize water to O_2_ due to unfavorable conduction band levels [13]. Co-doping strategies, such as with antimony, have been proposed to stabilize the oxidation state of Rh and suppress the formation of recombination centers, thereby enhancing O_2_ evolution activity [14].

Barium titanate (BaTiO_3_), a ferroelectric perovskite with a band gap of 3.2–3.4 eV, shares a similar band structure with SrTiO_3_ and has been widely studied for dielectric, piezoelectric, and optical applications [10,15]. Its photocatalytic potential, however, remains less explored. While undoped BaTiO_3_ is inefficient for solar harvesting due to its wide band gap, doping with elements such as Fe, Mo, Eu, and Rh has been shown to enhance its electronic and photocatalytic properties [16,17,18,19]. Rh doping, in particular, has been suggested to form mid-gap states that reduce the band gap without introducing recombination centers [19]. Despite these promising indications, studies on Rh-modified BaTiO_3_ remain limited. Maeda et al. reported p-type behavior in Rh-doped BaTiO_3_, but the observed cathodic photocurrent was low, indicating the need for further optimization and mechanistic understanding [8]. In addition to hydrogen production, Rh-doped BaTiO_3_ has potential for photocatalytic degradation of organic pollutants. While SrTiO_3_:Rh has been investigated for methylene blue degradation and thermoelectric applications [20,21], the photocatalytic efficiency of Rh-doped BaTiO_3_ has primarily been evaluated for hydrogen generation [8,9]. Given the tunability of BaTiO_3_’s crystal and electronic structure, a systematic investigation of Rh doping—its site occupancy, oxidation state, and effect on band structure—is warranted to fully assess its photocatalytic potential.

Beyond static electronic structure modifications, the photocatalytic performance of a material is fundamentally governed by the dynamics of photogenerated charge carriers. Upon light absorption, semiconductors generate electron–hole pairs on femtosecond timescales. These nonequilibrium carriers subsequently undergo relaxation, trapping, and recombination processes spanning femtoseconds to microseconds. The lifetimes of these excited states critically determine the probability that carriers will reach the surface and participate in redox reactions before recombining. Prolonged carrier lifetimes enhance photocatalytic activity, as demonstrated in various material systems where suppressed recombination correlates with increased H_2_ evolution or pollutant degradation. For instance, the formation of shallow trap states can double carrier lifetimes and boost H_2_ generation by nearly an order of magnitude, whereas deep traps typically act as recombination centers and degrade performance. Similarly, heterojunction engineering has been shown to extend carrier lifetimes by promoting spatial separation of electrons and holes. These findings underscore that optimizing charge carrier dynamics is as important as tuning the band gap for achieving high photocatalytic efficiency.

Despite the recognized importance of charge carrier dynamics, the nonequilibrium behavior of photogenerated carriers in Rh-doped BaTiO_3_ remains largely unexplored. The extent to which Rh doping influences carrier lifetimes, introduces beneficial or detrimental trap states, and affects the competition between surface reaction kinetics and recombination has not been systematically addressed. Understanding these dynamics is essential for rational design of Rh-doped BaTiO_3_ photocatalysts with enhanced performance.

Recently, we investigated the potential of modified barium titanate for the development of efficient water oxidation electrocatalysts using first-principles calculations [22]. Our findings demonstrate that Rh doping offers a dual benefit: it extends the light absorption of BaTiO_3_ across the entire visible spectrum and simultaneously reduces the overpotential required for water oxidation. Specifically, the TiO_2_-terminated BaTiO_3_ (001) surface was identified as the most catalytically promising orientation. It was shown that the aqueous environment plays a critical role in modulating the electrochemical performance of Rh-doped BaTiO_3_. This effect of improved surface reactivity establishes Rh-modified BaTiO_3_ as a promising photoanode material for PEC water-splitting systems.

The efficiency of photocatalytic hydrogen production is determined by the competition between electron transfer to protons and non-productive carrier loss processes. Upon photoexcitation, electron-hole pairs undergo ultrafast intra-band relaxation, which reduces the redox potential of electrons below the level required for H^+^ reduction, while subsequent recombination limits the lifetime of carriers available for reaction at the catalyst surface.

First-principles prediction and interpretation of pathways of excited state dynamics is based on a range of approaches beyond born-Oppenheimer approximation. Such approaches explicitly model coupling between nuclear and electronic degrees of freedom and implement a concept of an open quantum system. Such approaches allow dissipation of electronic excitation energy into lattice vibrations.

In this work, we investigate the relaxation dynamics of electronic excitations on the (001) crystallographic surface of BaTiO_3_ modified with rhodium sites, which act as a nanocatalyst and introduce additional electrons into the system. The mechanisms of electron–hole relaxation under aqueous conditions are examined in detail. Based on these findings, we predict time-resolved spectra and frequency-dependent response functions that could be measured in ultrafast nonlinear time-resolved experiments. Such insights are crucial for the rational design of materials for photocatalytic water splitting and solar energy conversion. Our results reveal that optical excitation generates electron–hole pairs, in which the electron relaxes to the conduction band edge and subsequently localizes at the Rh site, while the hole migrates to the Rh site at a faster rate. This asymmetry in relaxation dynamics explains the significant enhancement in water decomposition efficiency upon rhodium doping. Furthermore, the calculated photoluminescence spectra exhibit distinct features depending on whether Rh ions are located on the surface or within the slab. This difference offers a potential experimental route to quantify the fraction of catalytically active surface sites relative to the total dopant concentration.

## 2. Results

**Figure 1:**
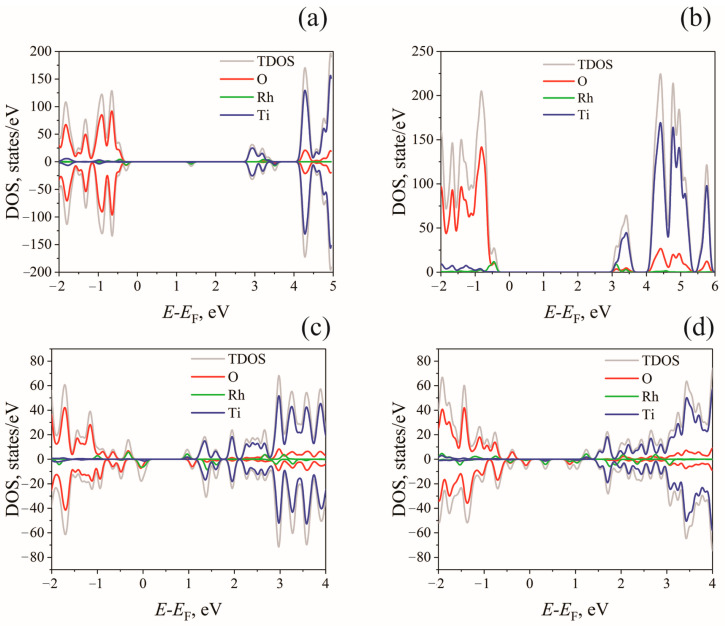
Total and partial densities of states for (**a**) **Rh@Ti-B** and (**b**) **Rh@Ba-B** structures. (**c**) Dry and (**d**) wet **Rh@Ti-S** structures. *E*_F_ is the Fermi energy.

*Density of states.* Figure 1 presents the results of density of states (DOS) calculations performed using the hybrid HSE06 functional. In all considered cases of Ti^4+^ substitution by Rh^4+^, doping leads to the appearance of states within the band gap. Figure 1a shows the results for the **Rh@Ti-B** structure. Substituting Ti^4+^ with Rh^4+^ introduces an additional level in the middle of the band gap. In the case of substitution of Ba^2+^ by Rh^3+^, the band gap width remains unchanged, and the Rh-4d electronic levels are located near the conduction band minimum (CBM) and valence band maximum (VBM). Accounting for the influence of an aqueous environment modifies the DOS within the band gap, which in turn affects the rates of nonadiabatic relaxation and recombination.

**Figure 2:**
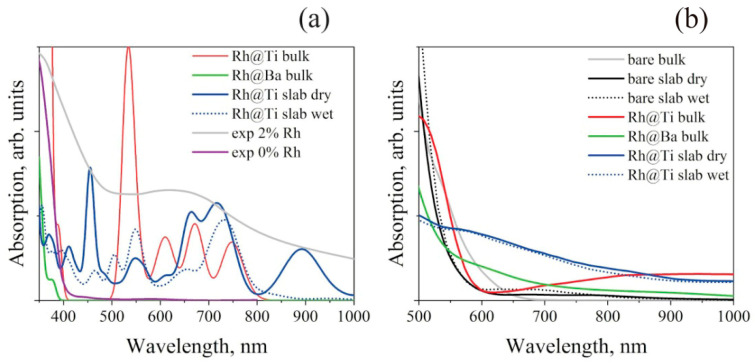
Optical absorption spectra of undoped and Rh-doped bulk and TiO_2_-terminated slabs. Both dry and wet surfaces are considered. (**a**) HSE06-calculated spectra for static lattices, compared with experimental data [25]. (**b**) Optical absorption spectra averaged along MD trajectories and calculated using the PBE+U functional at *T* = 300 K.

*Optical absorption.* Previously, we simulated the optical properties of the tetragonal phase of BaTiO_3_ [23]. It was shown that the optical absorption spectra calculated using the HSE06 functional with the inclusion of dynamic contributions are in good agreement with experimental data [7,24]. Rhodium doping of BaTiO_3_ nanoparticles induces absorption in the visible range [25], which is confirmed by HSE06 calculations (Figure 2a) [22]. The main difference from the experimentally measured spectra is the oscillatory behavior of the calculated absorption spectra, whereas the experimental spectra are monotonic functions.

Figure 2b shows the optical absorption spectra averaged over molecular dynamics (MD) trajectories at a temperature of 300 K. Since the calculations were performed using the PBE+*U* functional, all obtained spectra are red-shifted. The averaging eliminates oscillations in the spectra. This is because, as shown previously [23,26], thermal atomic motion alters the oscillator strengths and optical transition energies, thereby determining the shape of the optical absorption spectra.

The experimental absorption spectrum for Rh-doped BaTiO_3_ is a monotonically decreasing function with a distinct feature: as the wavelength increases to 500 nm, there is a slight rise-up to approximately 650 nm, after which the spectrum becomes steadily decreasing again. Theoretical calculations predict a monotonic decrease in optical absorption across the entire wavelength range considered for all models except the **Rh@Ti-B** structure. In this case, the absorption spectrum rapidly decreases up to 600 nm, then slowly increases, reaching a plateau at wavelengths beyond 850 nm.

This result indicates that the experimentally observed spectrum is a superposition of contributions. The feature in the experimental spectrum arises from configurations where the Rh^4+^ ion substitutes a Ti^4+^ ion within the bulk of the sample. It should also be noted that for spectra averaged along MD trajectories, the differences in absorption between dry and wet surfaces disappear.

**Figure 3:**
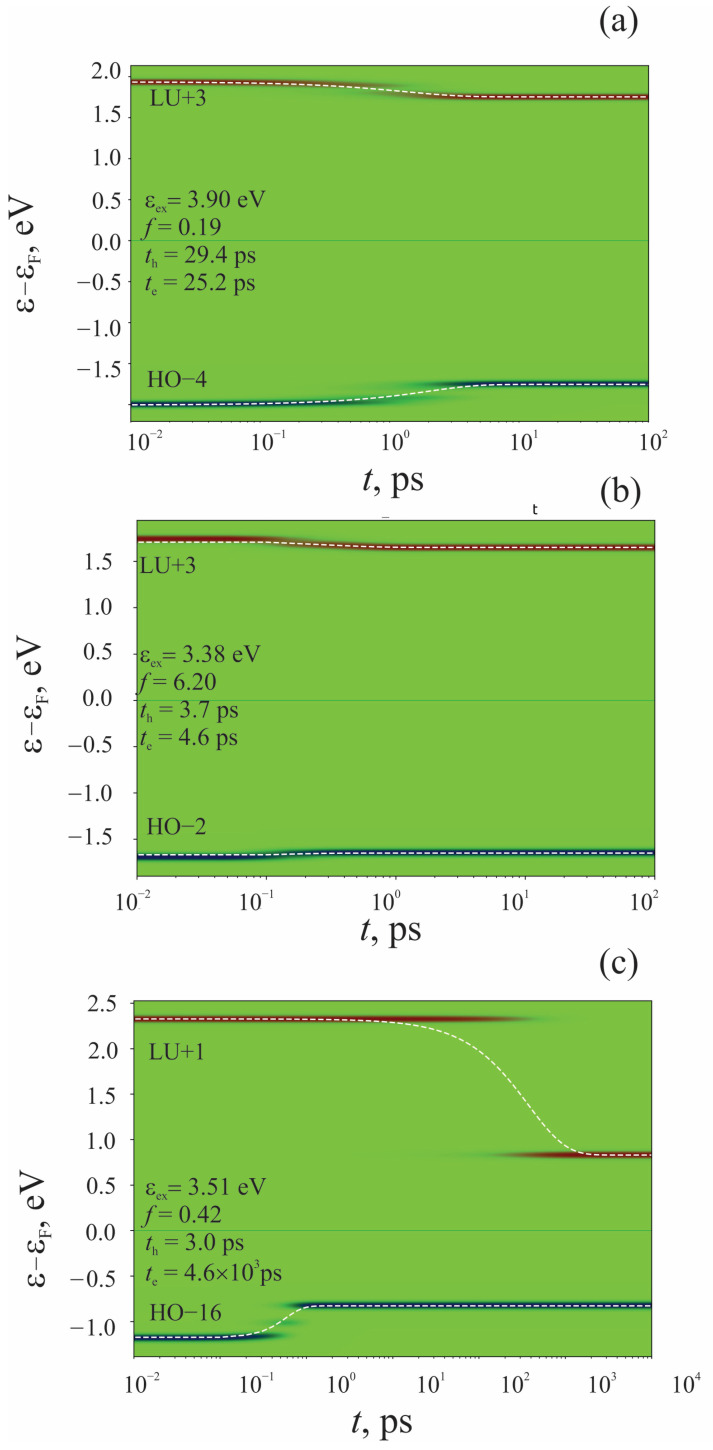
Nonadiabatic relaxation dynamics of photoexcited charges carriers in (**a**) **Rh@Ba-B** structure and (**b**) α- and (**c**) β-states of **Rh@Ti-B**. The initial excitation energies and oscillator strengths, as well as the relaxation times of excited electrons (*t_e_*) to the bottom of the band gap and holes (*t*_h_) to the top of the valence band are indicated on the corresponding panels. The red and blue colors represent the gain in population of electrons and holes, respectively. The green color indicates no change, which corresponds to the ground-state charge density. *ε*_F_ is the Fermi energy.

*Non-radiative relaxation.* Thermal motion of nuclei violates orthogonality of electronic states. The degree of this “violation” serves as a quantifiable measure of transition probability between two electronic states. The higher the temperature, the faster the nonadiabatic couplings. In the present Rh-doped BaTiO_3_ models, the dominant nonradiative pathways are phonon-assisted electronic transitions mediated by nonadiabatic electron–phonon coupling. Please see the Methods section for details. The properly processed transition rates enable the computation of excited state dynamics: Figure 3 illustrates the supercell calculated excited-state dynamics of electron–hole pairs in the **Rh@Ba-B** structure (Figure 3a) and of electron–hole pairs for the α (spin-up) and β (spin-down) states in the **Rh@Ti-B** structure (Figure 3b,c). The results were obtained by processing nonadiabatic couplings from adiabatic MD trajectories using spin-restricted and spin-unrestricted DFT, correspondingly. Bright optical transitions in the ultraviolet range are considered. Excitation energies, oscillator strengths, and relaxation times of electrons to the bottom of the conduction band (lowest occupied state, LU) and to the top of the valence band (highest occupied state, HO) are indicated as labels in the corresponding figure.

For the **Rh@Ba-B** structure, electrons and holes relax at approximately the same rate, with relaxation times of 29.4 ps and 25.2 ps, respectively. For electronic excitations of the α states in the **Rh@Ti-B** structure, relaxation occurs qualitatively similarly, with hole and electron relaxation times of 3.7 ps and 4.6 ps, respectively. In the case of the β states of electrons in the **Rh@Ti-B** structure, the lowest unoccupied conduction band minimum (LU) state at the middle of the band gap is separated by a large energy gap from the LU+1 state, leading to a sharp increase in the relaxation time of the excited electron to 4.6 ns. β holes relax to the HO state in 3 ps.

**Figure 4:**
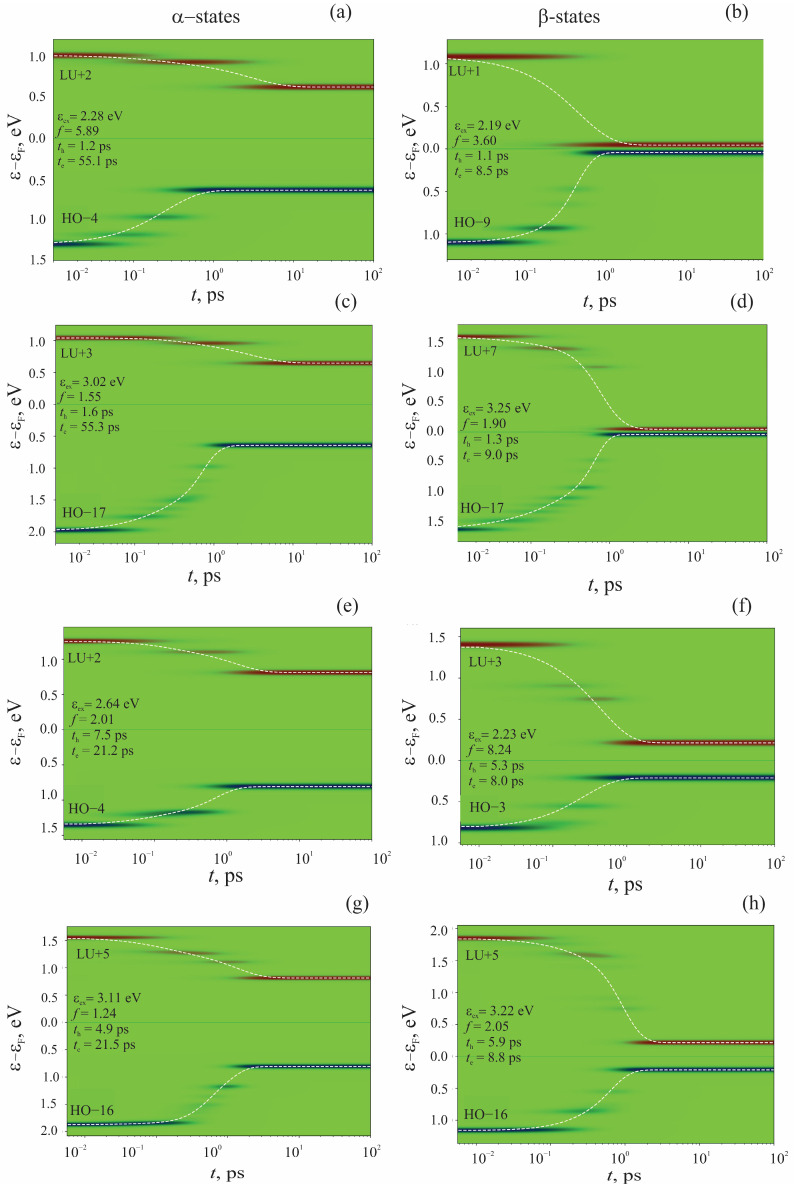
Nonadiabatic relaxation dynamics of photoexcited charge carriers on Rh-doped (001) BaTiO_3_ surfaces: (**a**–**d**) dry and (**e**–**h**) wet. Initial excitations are considered for absorbed light wavelengths in the green (2.19–2.64 eV) and ultraviolet (3.02–3.25 eV) ranges. The nonequilibrium relaxation of α (**left column**) and β (**right column**) states is analyzed. The initial excitation energies, pair of orbitals perturbed by excitation, and oscillator strengths, as well as the relaxation times of excited electrons (t_e_) to the bottom of the conduction band and holes (t_h_) to the top of the valence band are indicated on the corresponding panels. ε_F_ denotes the Fermi energy.

Figure 4 shows the excited-state dynamics of electron–hole pairs in dry (Figure 4a–d) and wet (Figure 4e–h) **Rh@Ti-B** structures. Initial excitations resulting from bright optical transitions in the visible (ε_ex_ ~ 2.19–2.64 eV) and ultraviolet (ε_ex_ ~ 3.02–3.25 eV) ranges are considered, for the dry **Rh@Ti-B** structure.

Table 1 summarizes the calculated non-radiative and radiative recombination parameters, along with the corresponding photoluminescence quantum yield (PLQY), for the α and β electronic states in three considered Rh-doped BaTiO_3_ configurations. These data provide critical insights into how doping site, localization, and solvation affect the fate of photogenerated charge carriers.

*Non-Radiative Recombination.* The non-radiative recombination rate (*k*_NRR_) and lifetime (*τ*_NRR_) are derived from Redfield theory, reflecting phonon-mediated electron–hole annihilation. For **Rh@Ti-B**, the α state exhibits a moderate *k*_NRR_ of 2.7 × 10^−3^ ps^−1^, corresponding to a *τ*_NRR_ of 370 ps. In contrast, the β state shows a significantly faster non-radiative decay (6.7 × 10^−2^ ps^−1^, *τ*_NRR_ = 15 ps), indicating that β-state excitations are more strongly coupled to the lattice and recombine non-radiatively an order of magnitude faster. **Rh@Ba-B** displays a single relaxation channel with parameters similar to the α state of **Rh@Ti-B** (*τ*_NRR_ ≈ 380 ps), suggesting that Ba-site doping introduces mid-gap states with comparable electron–phonon coupling strength.

Dramatically different behavior is observed for the **Rh@Ti-S** structures. Here, non-radiative recombination is ultrafast, with *k*_NRR_ values in the range of 2.5–9.5 ps^−1^ and *τ*_NRR_ as short as 0.1–0.4 ps that is from three to four orders of magnitude faster than in the bulk. This acceleration is attributed to the reduced coordination and enhanced lattice flexibility at the surface, which facilitates efficient energy dissipation. Wetting the surface further increases *k*_NRR_ (e.g., from 2.5 to 9.5 ps^−1^ for the α state), implying that the aqueous environment enhances non-radiative pathways, possibly through solvation-induced stabilization of intermediate states.

*Radiative Recombination.* Radiative recombination rates (*k*_RR_) are universally much smaller than non-radiative rates, confirming that photoluminescence is strongly quenched in these systems. In bulk **Rh@Ti-B**, *k*_RR_ ranges from 9.4 × 10^−4^ ps^−1^ (α) to 4.7 × 10^−4^ ps^−1^ (β), yielding long radiative lifetimes (*τ*_RR_) of 1.1 ns and 21 ns, respectively. Rh@Ba-B exhibits a somewhat higher *k*_RR_ (1.1 × 10^−2^ ps^−1^, *τ*_RR_ = 90 ps), indicating that Ba-site doping introduces a more radiatively permissive transition.

On the surface (**Rh@Ti-S**), radiative recombination becomes extremely slow: *k*_RR_ drops to 10^−5^–10^−7^ ps^−1^, with *τ*_RR_ reaching microseconds (0.037–4.4 μs). This dramatic suppression arises from the drastic reduction in the LU → HO transition energy and the small value of the associated oscillator strength. (See Equation (15) and Table 2).

*Photoluminescence Quantum Yield (PLQY).* The PLQY, defined as *k*_RR_/(*k*_RR_ + *k*_NRR_), quantifies the probability of radiative versus non-radiative decay. Bulk **Rh@Ti-B** gives low PLQY values of 0.034 (α) and 0.007 (β), while **Rh@Ba-B** achieves a modest PLQY of 0.16—the highest among all studied systems—consistent with its more balanced recombination kinetics.

In contrast, the **Rh@Ti-S** structures exhibit extremely low PLQY on the order of 10^−5^–10^−8^. The dry surface yields PLQY of ~10^−5^ for the α state and ~7 × 10^−8^ for the β state; wet conditions further reduce these values to ~3 × 10^−8^ and ~4 × 10^−8^, respectively. Such negligible quantum yields indicate that nearly all photogenerated excitons recombine non-radiatively, which, paradoxically, can be beneficial for photocatalysis: rapid non-radiative relaxation can drive charge separation and deliver hot carriers to reactive surface sites before recombination, as long as the recombination pathway does not compete with charge transfer to adsorbed species.

**Figure 5:**
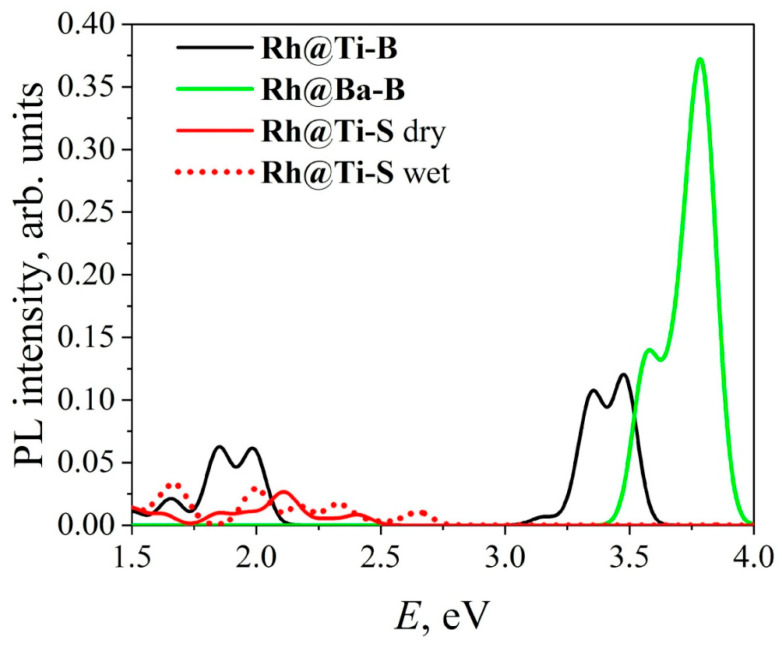
Time-integrated photoluminescence spectra for structures investigated.

*Time-integrated photoluminescence.* Time-integrated photoluminescence (PL) spectra of Rh-doped BaTiO_3_ for the four studied configurations. (Figure 5) The bulk **Rh@Ba-B** system exhibits a broad emission band centered around 3.5–3.8 eV, corresponding to the highest PL quantum yield among all systems. In contrast, **Rh@Ti-B** shows significantly suppressed emission in ultraviolet (3.1–3.6 eV) and visible (less than 2.2 eV) regions. Both dry and wet **Rh@Ti-S** surface structures display almost completely quenched photoluminescence across the entire range (energy less than 2.5 eV and 2.7 eV for dry and wet surfaces, correspondingly) with low intensity. The difference in PL intensity between bulk and surface configurations provides a potential experimental fingerprint to distinguish catalytically active surface Rh sites (PL-silent) from optically active bulk dopants (PL-active). There is an interesting observation that each of the computed spectra demonstrate a non-Kasha mechanism of emission. Specifically, each PL spectrum contains more than one peak. This is interpreted as the electronic excitation cascading down in energy and visiting quasi-stationary states with extended lifetime. Each of such long-lived quasi-stationary states provides a contribution to the integrated emission profile, in contrast to the idealistic image of Kasha mechanism of emission from the lowest singlet excitation. The rational explanation of these additional states originates from energy sub-gaps between discrete, isolated energy levels contributed by doping. Additional processes of optical transitions are available in the NIR range for the models with low energy gaps and sub-gaps. These transitions listed in Table A1 are beyond the traditional observation range in the UV-visible spectroscopy and are typically suppressed by a low signal-to-noise ratio. Those transitions are shown in Figure A2.

It is important to distinguish between the calculated band-edge PLQY values and the full emission spectrum. The low PLQY values (∼10^−8^) reported in Table 1 correspond strictly to emission from the lowest available excited states, in accordance with Kasha’s rule. However, the presence of significant sub-gap states allows for the violation of Kasha’s rule. Excitations can be trapped in quasi-stationary states during internal conversion, leading to non-Kasha luminescence at energies substantially above the fundamental gap. Our computational methodology captures this effect, predicting multiple emission lines across both the visible (Figure 5) and infrared regions (Figure A2). Experimentally, verifying these higher-energy infrared transitions remains challenging and typically requires specialized setups, such as cooled InGaAs detectors, to overcome low signal-to-noise ratios.

Efficient charge transfer is a fundamental prerequisite for photocatalysis. While direct experimental measurement of charge transfer dynamics is possible, it remains technically challenging and limited in scope. Alternatively, one can consider the initial photoexcitation as undergoing several competing, mutually exclusive relaxation pathways: radiative recombination (photoluminescence), productive charge transfer, and various non-radiative channels (e.g., phonon emission, trapping in metastable states, or undesired side reactions). Because the sum of the quantum yields of these competing pathways must equal unity, a decrease in one channel often corresponds to an increase in another. Consequently, the quenching of photoluminescence can serve as a qualitative, indirect indicator of efficient charge transfer. It should be noted that photoluminescence quenching alone does not distinguish productive charge separation from trap-assisted nonradiative recombination and therefore must be interpreted together with the calculated carrier relaxation pathways.

Although this inverse correlation is subject to system-specific limitations, it provides a valuable practical proxy.

In this context, photoluminescence (PL) spectroscopy offers a distinct advantage for the preliminary assessment of Rh-doped BaTiO_3_. As illustrated in Figure A2, the PL emission bands in the infrared (0–1.4 eV) and visible (2.1–2.7 eV) ranges are attributed to surface Rh^4+^ ions. Thus, PL measurements provide critical insights into the relative abundance of these active surface species, enabling a preliminary evaluation of the synthesized samples’ expected performance prior to practical deployment. Ultimately, PL spectroscopy can be leveraged as a robust, non-destructive quality control metric for the large-scale production of these photocatalysts.

## 3. Materials and Methods

### 3.1. Molecular Dynamics and Nonadiabatic Couplings

We employ the reduced density matrix (RDM) formalism based on Redfield theory to model nonadiabatic charge dynamics in the studied photocatalytic systems. Within this approach, the electronic subsystem is treated as an open quantum system interacting with lattice vibrations (phonon bath), where the time evolution of the electronic density matrix is governed by the Liouville–von Neumann equation with Redfield relaxation terms. The method combines density functional theory (DFT) calculations of electronic structure with on-the-fly computation of electron–phonon nonadiabatic couplings, allowing us to describe hot carrier relaxation, charge transfer, and recombination processes on equal footing. The autocorrelation functions of the interaction Hamiltonian provide state-to-state transition rates that enter into the equation of motion for electronic degrees of freedom, enabling the calculation of time-resolved observables including carrier lifetimes and energy dissipation dynamics.

To account for thermal effects, adiabatic molecular dynamics (MD) simulations were performed with a temperature of *T =* 300 K maintained by a Nosé–Hoover thermostat, satisfying

(1) $$\frac{3}{2}N^{ion}T=\frac{1}{2}\sum_{I=1}^{N^{ion}}M_{I}{\left(\frac{d{\vec{R}}_{I}}{dt}\right)}^{2}$$

where $M_{I}$ and ${\vec{R}}_{I}$ are the mass and position of ion *I*, respectively. Ionic motion is governed by Newton’s equations:

(2) $$\frac{d^{2}{\vec{R}}_{I}}{dt^{2}}=\frac{{\vec{F}}_{I}}{M_{I}},$$

solved using standard ab initio MD tools. This provided trajectories of nuclear positions ${\vec{R}}_{I}(t)$ and interatomic distances, which in turn influenced KS orbitals, orbital energies, transition dipoles, and oscillator strengths at each time step.

For adiabatic MD calculations, the geometry-optimized models were heated to the target temperature (300 K) using a Nose’–Hoover thermostat with repeated velocity rescaling. Then, 1 ps microcanonical trajectories were generated using the Verlet algorithm with a time step of 0.5 fs.

Nonadiabatic couplings (NACs) between electronic states were computed along the MD trajectory using the time-dependent overlap of KS wavefunctions:

(3) $$V_{\sigma ,ij}(t)=-\frac{i\hbar }{2∆t}\int d\vec{r}\phi_{\sigma i}^{KS*}\left(\vec{r},t\right)\phi_{\sigma j}^{KS}(\vec{r},t+∆t)+h.c.$$

The autocorrelation function of the NACs,

(4) $$M_{\sigma ,ijkl}=\frac{1}{T_{MD}}\int_{0}^{T_{MD}}V_{\sigma ,ij}\left(t+\tau \right)V_{\sigma ,kl}(t)dt,$$

captures the memory of the electron–nuclear interaction. Its Fourier transform yields the Redfield tensor components

(5) $$R_{\sigma ,ijkl}=\Gamma_{\sigma ,ljik}^{+}+\Gamma_{\sigma ,ljik}^{-}-\delta_{jl}\sum_{m}\Gamma_{immk}^{+}-\delta_{lk}\sum_{m}\Gamma_{jmml}^{-}$$

The partial rates $\Gamma^{\pm }$ are expressed in terms of autocorrelation functions *M* of the interaction Hamiltonian

(6) $$\Gamma_{ljik}^{+}=\int_{0}^{t}d\tau M_{\sigma ,ljik}(\tau )exp(-i\omega_{ik}\tau )$$

(7) $$\Gamma_{ljik}^{+}=\int_{0}^{t}d\tau M_{\sigma ,ljik}(-\tau )exp(-i\omega_{lj}\tau )$$

$\Gamma^{\pm }$ are proportional to the spectral density of the bath fluctuations, which is obtained by taking the Fourier transform of the autocorrelation function of the interaction Hamiltonian at the resonant transition frequencies.

Figure A3 shows representative examples of correlation function components *M*_ljik_(t) (Equation (4)). It is very important that the autocorrelation function element *M*_ljik_(t) decays within 1 fs, corresponding to a spectral density cutoff frequency of *f_c_* ≳ 0.7 eV (169 THz). This exceptionally short bath correlation time strongly supports the validity of the Markov approximation employed in our Redfield formalism.

$R_{\sigma ,ijkl}$ matrix elements enter the dissipative part of the quantum master equation for the reduced density matrix $\rho_{\sigma }$:

(8) $$\frac{d\rho_{\sigma ,ij}}{dt}=-\frac{i}{\hbar }\sum_{k}\left(F_{\sigma ,ik}\rho_{\sigma ,kj}-\rho_{\sigma ,ik}F_{\sigma ,kj}\right)+{\frac{d\rho_{\sigma ,ij}}{dt}|}_{diss},$$

with

(9) $${\frac{d\rho_{\sigma ,ij}}{dt}|}_{diss}=\sum_{kl}R_{\sigma ,ijkl}\rho_{\sigma ,kl},$$

Diagonal elements $\rho_{\sigma ,ii}\left(t\right)$ represent time-dependent orbital occupations, from which relaxation times for electrons and holes are extracted.

The relaxation times as all other time dependent observables are obtained in terms of solution of equation of motion (8)–(9) for the diagonal elements of reduced density matrix. $\rho_{\sigma ,ii}=\rho_{\sigma ,ii}(t)$. These solutions are utilized to construct multiple observables of interest such as nonequilibrium distribution of charge in energy and time Δn(ε,t), expectation values of energy of a carrier ${\langle E\rangle }_{e}\left(t\right)$, ${\langle E\rangle }_{h}\left(t\right)$, rates of energy dissipation for a carrier, and PL spectra. Details can be found, for example, in Ref. [27].

Briefly, the difference in nonequilibrium distribution and the equilibrium $n_{\sigma ,i}^{equilibrium}\left(\varepsilon \right)$ distribution provides the comprehensive explanation of electron and hole dynamics as a function of energy and time. Evolution of electrons is obtained by screening orbitals with indices i≥LU. Evolution of holes is obtained by screening of orbitals with i≤HO.

(10) $$\Delta n_{\sigma ,e/h}\left(\varepsilon ,t\right)=-n_{\sigma ,i}^{equilibrium}\left(\varepsilon \right)+\sum_{i\geq LU\;/i\leq HO}\delta \left(\varepsilon -\varepsilon_{i,\sigma }\left(t\right)\right)\rho_{\sigma ,ii}\left(t\right)$$

This equation describes the dynamics of a population gain when Δn > 0 and a population loss when Δn < 0 at energy ε, which corresponds to the electron and the hole parts of an excitation. The expectation energy of a photoexcited electron can be expressed for a chosen spin projection as follows:

(11) $$\langle \Delta \varepsilon_{\sigma }^{e}\rangle \left(t\right)=\sum_{i\geq {LU}_{\sigma }}\rho_{\sigma ,ii}\left(t\right)\varepsilon_{\sigma }^{e}(t)$$

(12) $$\langle E_{e,\sigma }\rangle \left(t\right)=\frac{\langle \Delta \varepsilon_{\sigma }^{e}\rangle \left(t\right)-\langle \Delta \varepsilon_{\sigma }^{e}\rangle \left(\infty \right)}{\langle \Delta \varepsilon_{\sigma }^{e}\rangle \left(0\right)-\langle \Delta \varepsilon_{\sigma }^{e}\rangle \left(\infty \right)}$$

Calculated relaxation time *τ_e_* and relaxation rate *k_e_* for electrons and holes are computed as:

(13) $$\tau_{e}=k_{e}^{-1}=\int_{0}^{\infty }\langle E_{e,\sigma }\rangle \left(t\right)dt$$

The expectation values of a photoexcited hole are defined analogously.

The computational methods used in this study, based on the reduced density matrix formalism within the framework of Redfield theory [28,29], have been detailed in our previous works. Specifically, the general concept is described in Ref. [30], the implementation of nonadiabatic couplings—including the treatment of open-shell systems—is detailed in Refs. [27,31], and the ab initio calculation of PL spectra is presented in Refs. [32,33].

### 3.2. Recombination Lifetime

#### 3.2.1. Radiative Recombination Rate (RR)

The radiative recombination rate is calculated based on the Einstein coefficients for spontaneous emission [34]. The oscillator strength for an electronic transition from the conduction band minimum (state 2) to the valence band maximum (state 1) is defined as:

(14) $$f_{21}=\frac{2m\nu_{21}}{3e^{2}h}{\left|D_{21}\right|}^{2}$$

where m is the electron mass, *h* is the Planck constant, *e* is the elementary charge, ν_21_ is the transition frequency, and $D_{21}=e\left\langle \psi_{1}^{KS}|r|\psi_{2}^{KS}\right\rangle$ denotes the transition dipole moment matrix element calculated from Kohn–Sham orbitals. The radiative rate is then obtained as:

(15) $$k_{RR}=\frac{8\pi^{2}\nu_{21}^{2}e^{2}}{mc^{3}}\frac{g_{1}}{g_{2}}f_{21}$$

where $g_{1}$ and $g_{2}$ are the degeneracies of electronic states and *c* is the speed of light. The radiative lifetime is defined as $\tau_{RR}=k_{RR}^{-1}$. Note that non-radiative recombination pathways (e.g., defect- or phonon-mediated processes) typically dominate carrier dynamics in real photocatalysts, and the computed radiative lifetimes represent an upper-bound estimate.

#### 3.2.2. Nonradiative Recombination Rate (NRR)

The term *R*_2211_ represents the population transfer rate from state 2 to state 1. For a specific non-radiative recombination transition from the conduction band minimum to the valence band maximum, the recombination rate constant is given directly by the corresponding diagonal element of the Redfield tensor $k_{NRR}=R_{2211}$. The nonradiative lifetime is defined as $\tau_{NRR}=k_{NRR}^{-1}$.

### 3.3. Photoluminescence

The time-resolved emission spectrum at an instant of time is obtained according to the following equation:

(16) $$E_{\sigma }\left(\varepsilon ,t\right)=\sum_{ij}f_{\sigma ,ij}\delta (\varepsilon -\varepsilon_{\sigma ,ij})\left(\rho_{\sigma ,jj}\left(t\right)-\rho_{\sigma ,ii}\left(t\right)\right),$$

$\varepsilon_{\sigma ij}$ and $f_{\sigma ,ij}$ are the transition energy and oscillator strength, correspondingly. $\rho_{\sigma ,jj}$ and $\rho_{\sigma ,ii}$ are the populations of *j*th and *i*th states. The time-integrated emission spectrum is calculated by the integration of the time-resolved emission over the duration of the trajectory *T*_MD_. $E\left(\varepsilon \right)=E_{\alpha }\left(\varepsilon \right)+E_{\beta }\left(\varepsilon \right)$

(17) $$E_{\sigma }\left(\varepsilon \right)=\frac{1}{T_{MD}}\int_{0}^{T_{MD}}E(\varepsilon ,t)dt,$$

### 3.4. Computation Details

#### 3.4.1. Electronic Structure Calculations

**Figure A1:**
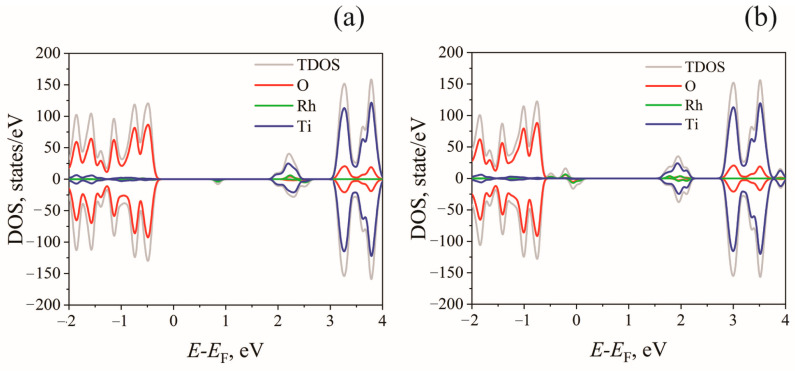
(**a**) PBE+*U* and (**b**) PBE calculated electronic DOS for **Rh@Ti-B** structure.

**Figure A2:**
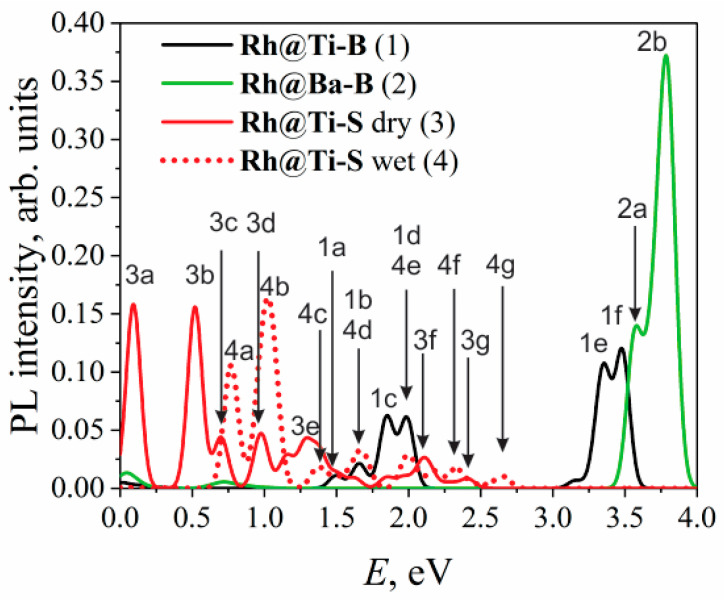
Complete range of optical transitions including near-infrared transitions. Peak labels indicate model and spectral transitions as listed in Table A1.

**Figure A3:**
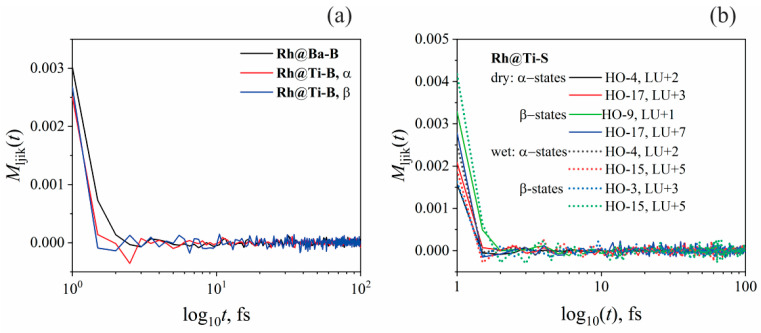
Autocorrelation functions *M*_ljik_ (Equation (4)) for the (**a**) bulk and (**b**) slab models. Results are shown for the same electronic excitations as in Figure 3 and Figure 4.

The electronic structure—including Coulomb, correlation, and exchange interactions among electrons, as well as electron–ion interactions—was calculated using DFT by self-consistently solving the Kohn–Sham equations, as implemented in the Vienna Ab Initio Simulation Package (VASP) [35,36]. The calculations employed the Heyd–Scuseria–Ernzerhof (HSE06) hybrid functional [37] and the Perdew–Burke–Ernzerhof (PBE) exchange-correlation functional within the generalized gradient approximation [38]. For the HSE06 functional, the exact Hartree–Fock exchange contribution was set using a mixing parameter of 0.25. The Dudarev approach [39] was used to treat the on-site Coulomb correlations with a *U-J* value of 2.6 eV for Ti-3d electrons, and with a *U-J* value of 5.5 eV for Rh-4d electrons when applying the PBE functional [40]. Choosing the *U−J* parameter equal to 5.5 eV in the PBE+U calculations ensures that the relative position of this level with respect to the conduction band minimum (CBM) and valence band maximum (VBM) matches that obtained with the HSE06 functional (Figure A1). The projector augmented-wave method [41], based on the pseudopotential concept within a plane-wave basis set, was used to perform the calculations. All molecular dynamics simulations were carried out using the PBE+*U* functional at a temperature of 300 K, with temperature control implemented via the Nosé–Hoover thermostat [42]. The calculations employed a plane-wave cutoff energy of 500 eV. Postprocessing of the VASP output data was carried out using the VASPKIT code [43].

#### 3.4.2. Structure Models

**Figure 6:**
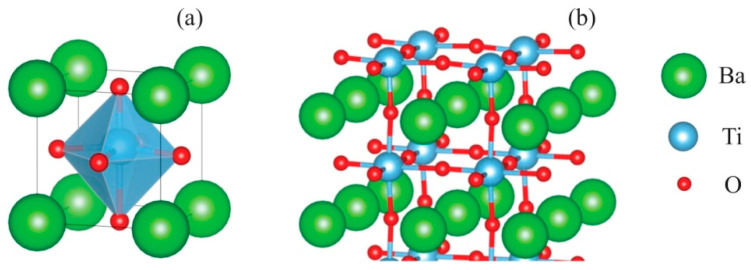
(**a**) Crystal structure of tetragonal BaTiO_3_, (**b**) TiO_2_-terminated (001) surfaces of tetragonal BaTiO_3_. Green, cyan, and red speheres stand for Ba, Ti, O ions, respectively.

In this study, the modeling was performed using the tetragonal phase of BaTiO_3_ (space group *P*4*mm*, #99, Figure 6a) which is not the ground state at *T* = 0 K but is stable at room temperature. The initial crystal structure was taken from the Materials Project database [44]. A 3 × 3 × 3 supercell of the primitive cell, consisting of 135 atoms, was used in the case of bulk structure modeling. To model Rh-doped BaTiO_3_, we substituted Ti^4+^ (structure **Rh@Ti-B**) and Ba^2+^ (structure **Rh@Ba-B**) ions with Rh atoms in the crystal structure. Since experimental studies have detected Rh^3+^ and Rh^4+^ in doped BaTiO_3_ [25], when doping is achieved by substituting Ba ions with Rh, one electron was removed to maintain the rhodium ion in the +3 oxidation state. During the modeling process, a singlet spin state was maintained for Rh^3+^ and a doublet spin state for Rh^4+^ [45].

To construct the surface models, (001)-oriented BaTiO_3_ slabs were created, consisting of eleven symmetric TiO_2_ and BaO layers with respect to the mirror plane. The slab has TiO_2_ terminations and comprised 112 atoms. The (001) orientation was selected because it represents the most stable structure (Figure 6b) [46]. A 15 Å thick vacuum layer was introduced perpendicular to the slabs to prevent spurious interactions between periodic images. This non-stoichiometric slab remains symmetric when substituting Ti atoms with Rh in the outermost layer (structure **Rh@Ti-S**), thereby avoiding the formation of a dipole moment—an effect that could otherwise significantly distort the calculated energies under periodic boundary conditions. This work focuses on the TiO_2_-terminated slab, as the BaO-terminated surface has recently been reported to be unstable under operating conditions [47]. The thermodynamic corrections for the solvation effect were calculated using VASPsol [48]. It allowed us to consider surface wetting through the water continuum model and distinguish dry and wet conditions. Because the VASPsol approach represents water as a dielectric continuum, explicit solvent vibrational modes, including high-frequency O–H stretching motions, are not included. Consequently, the calculated nonadiabatic relaxation rates for wet surfaces should be interpreted as describing solvation effects without explicit solvent dynamics. If the continuum model applied, the wet conditions are stated. The practical implementation of the open quantum system concept in atomistic simulation has several foundations. The nonadiabatic couplings along the adiabatic MD trajectory increases computational efficiency and allows to explore transitions beyond a harmonic regime [49]. The atomistic data on nonadiabatic couplings can be used to propagate an electronic state by a range of methods [50], from multiple spawning [51] to surface hopping [52,53,54,55,56], and density matrix propagation. The Redfield formulation allows for bath-assisted dynamics of electronic degrees of freedom [28,29]. The practical implementations of Redfield formulation in basis of Kohn–Sham orbitals was verified on a range of applications to surfaces, interfaces, and nanomaterials, such as silver to silicon nanointerface [30], doped titania [27,31], and perovskite nanostructures [57].

## 4. Conclusions

Understanding the interplay between electronic structure, charge carrier dynamics, and recombination mechanisms is essential for designing efficient photocatalysts for solar water splitting. In this work, we present a first-principles study of Rh-doped BaTiO_3_, combining DFT with the HSE06 hybrid functional and nonadiabatic charge dynamics within Redfield theory formalism. We systematically investigate bulk and (001) surface models with Rh substituting Ti^4+^ under dry and wet conditions. Our results show that Rh doping introduces mid-gap states that extend optical absorption into the visible range, in agreement with experimental spectra. Nonadiabatic relaxation dynamics reveal strong site-dependent behavior: in bulk **Rh@Ti-B**, two distinct relaxation channels (α and β) are identified, with electron relaxation times ranging from 3.7 ps to 4.6 ns depending on the orbital character and energy gap. For Rh@Ba-B, electrons and holes relax at similar rates (~25–30 ps). Remarkably, for **Rh@Ti-S,** model non-radiative recombination becomes ultrafast (τ_NRR_ = 0.1–0.4 ps), while radiative recombination is suppressed by several orders of magnitude (τ_RR_ up to microseconds), leading to extremely low photoluminescence quantum yields (PLQY~10^−5^–10^−8^). Wetting the surface further accelerates non-radiative decay. These findings demonstrate that Rh-doped BaTiO_3_, particularly the TiO_2_-terminated (001) surface, promotes efficient spatial separation of photogenerated charges and rapid carrier thermalization, which are key prerequisites for photocatalytic water splitting, despite strong photoluminescence quenching.

## Figures and Tables

**Table 1:** Non-radiative recombination rate (*k*_NRR_), non-radiative recombination time (*τ*_NRR_), radiative recombination rate (*k*_RR_), radiative recombination time (*τ*_RR_) and photoluminescence quantum yield (PLQY) for α and β states for structures investigated.

Structure	*k*_NRR_, ps^−1^		*τ*_NRR_, ps		*k*_RR_, ps^−1^		*τ*_RR_, ps		PLQY	
	α	β	α	β	α	β	α	β	α	β
**Rh@Ti-B**	2.7 × 10^−3^	6.7 × 10^−2^	3.7 × 10^2^	15.0	9.4 × 10^−4^	4.7 × 10^−4^	1.1 × 10^3^	2.1 × 10^4^	3.4 × 10^−2^	7 × 10^−3^
**Rh@Ba-B**	2.6 × 10^−3^	–	3.8 × 10^2^	–	1.1 × 10^−2^	–	9.0 × 10^3^	–	0.16	–
**Rh@Ti-S** dry	2.5	3.2	0.4	0.3	2.7 × 10^−5^	2.2 × 10^−7^	3.7 × 10^4^	4.4 × 10^6^	10^−5^	7 × 10^−8^
**Rh@Ti-S** wet	9.5	5.4	0.1	0.2	2.4 × 10^−5^	2.3 × 10^−7^	4.1 × 10^5^	4.3 × 10^6^	3 × 10^−8^	4 × 10^−8^

**Table 2:** Parameters for spontaneous emission of structures considered.

Structure	*ε = hν*_21_	g_1_	g_2_	*f*_21_
**Rh@Ba-B**	3.51	1	1	0.21
**Rh@Ti-B**				
α-state	3.30	2	1	1.00
β-state	1.66	2	1	1.99
**Rh@Ti-S** dry				
α-state	1.29	1	2	0.19
β-state	0.09	1	1	0.70
**Rh@Ti-S** wet				
α-state	1.62	1	1	0.02
β-state	0.42	1	1	0.03

**Figure A4:**
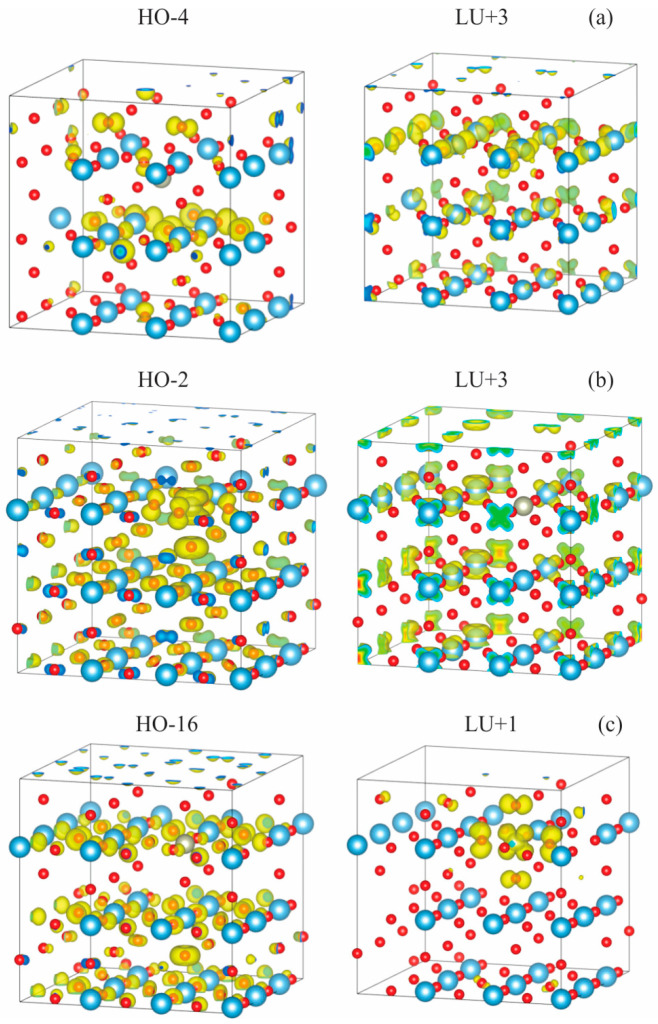
Partial charge densities of the orbitals involved in the initial excitations shown in Figure 3. Panels (**a**–**c**) illustrate pairs of electron hole states contributing to representative excitations. Each transition is labeled as *HO-i* → *LU+j* to symbolize electron promotion from occupied orbital *HO-i* into unoccupied orbital *LU+j*, as follows. (**a**) HO-4 → LU+3. (**b**) HO-2 → LU+3. (**c**) HO-16 → LU+1.

**Figure A5:**
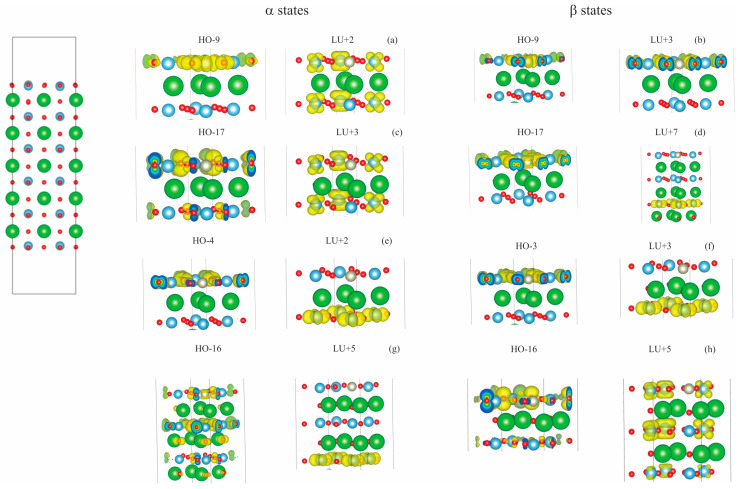
Partial charge densities of the orbitals involved in the initial excitations shown in Figure 4. Panels (**a**–**h**) illustrate pairs of electron hole states contributing to representative excitations. Each transition is labeled as *HO_α__/__β_-i* → *LU_α__/__β_+j* to symbolize electron promotion from occupied orbital *HO_α__/__β_-i* into unoccupied orbital *LU_α__/__β_+j*. Note that transitions for spin majority *α* (**a**,**c**,**e**,**g**) and spin minority *β* components (**b**,**d**,**f**,**h**) are independent and not coinciding, as revealed in visual patterns of iso-surfaces. (**a**) HO_α_-9 → LU_α_+2. (**b**) HO_β_-9 → LU_β_+3. (**c**) HO_α_-17 → LU_α_+3. (**d**) HO_β_-17 → LU_β_+7. (**e**) HO_α_-4 → LU_α_+2. (**f**) HO_β_-3 → LU_β_+3. (**g**) HO_α_-16 → LU_α_+5. (**h**) HO_β_-16 → LU_β_+5. Green, cyan, grey, and red spheres stand for Ba, Ti, Rh, O ions, respectively.

**Figure A6:**
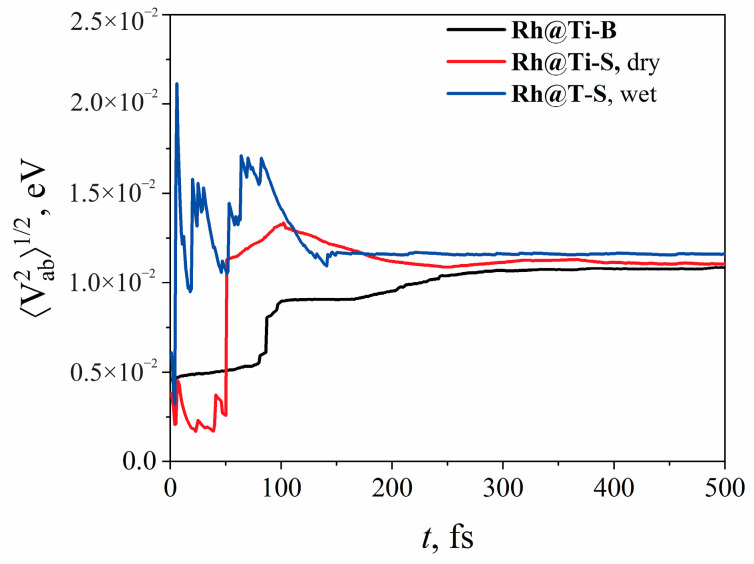
Convergence of average nonadiabatic coupling ${\langle V_{ab}^{2}\rangle }^{1/2}$ as a function of number of time steps in Rh@Ti-B, and dry and wet Rh@Ti-S structures. *a* and *b* refer to HO-3 and HO-2 states for α-electrons.

## Data Availability

The original contributions presented in this study are included in the article. Further inquiries can be directed to the corresponding authors.

## Abbreviations

The following abbreviations are used in this manuscript:

DFT	Density Functional Theory
MD	Molecular dynamics
DOS	Density of states
PLQY	Photoluminescence quantum yield
RDM	Reduced density matrix
VBM	Valence band maximum
CBM	Conduction band minimum

## Appendix A

To determine the optimal value of the *U-J* parameter within the DFT+U framework, we performed a systematic fitting to reproduce the electronic structure calculated using the HSE06 hybrid functional. Figure A1a presents the calculated electronic DOS for the **Rh@Ti-B** structure using the U−J parameter of 5.5 eV for the Rh-4d states. The impurity level within the band gap is located at the same position relative to the VBM and CBM as that obtained from calculations using the HSE06 functional. PBE calculations without a *U-J* parameter on Rh-4d states incorrectly places Rh impurity states near the valence band top in both α- and β-states (Figure A1b). Optimal DFT+*U* induces a distinct, mid-gap impurity peak formed exclusively by spin-down Rh states and restores the correct spin splitting.

Table A1 summarizes the parameters of the PL peaks shown in Figure A2. All observed transitions are inter-band, except for the lowest-energy feature in the **Rh@Ti-B** structure. This 1.5 eV peak originates from an intra-band transition from the conduction band minimum to a mid-gap impurity level (Figure 1a). Additionally, due to their low PL intensity, peaks in the **Rh@Ba-B** structure associated with near-zero-energy intra-band conduction-band transitions and ~0.7 eV valence-band transitions are omitted from Figure 5 and Table A1.

**Table A1:** Parameters of the PL spectra peaks marked in Figure 5. Arrow symbolizes transition with loss of energy from higher energy state to lower energy state, releasing excess of energy in form of an emitted photon.

Structure	PL Peak	Energy, eV	Transition	Spin State
**Rh@Ti-B**	1a	1.50	LU+1 → LU	β
	1b	1.66	LU → HO-1	β
	1c	1.85	LU → HO-2–HO-4	β
	1d	1.98	LU → HO-5–HO-10	β
	1e	3.35	LU → HO-1–HO-4	α
	1f	3.50	LU → HO-5–HO-10	α
**Rh@Ba-B**	2a	3.57	LU+1 → HO-1	–
	2b	3.67	LU → HO-2	–
**Rh@Ti-S** dry	3a	0.09	LU → HO-1	β
	3b	0.52	LU → HO-1, HO-2	β
	3c	0.70	LU → HO-4	β
	3d	0.97	LU → HO-5, HO-6	β
	3e	1.27	LU → HO-12, HO-13	β
	3f	2.17	LU → HO-8, HO-9	α
	3g	2.40	LU → HO-15	α
**Rh@Ti-S** wet	3a	0.77	LU → HO-1	β
	3b	0.97	LU → HO-2	β
	3c	1.37	LU → HO-1, HO-6	β
	3d	1.62	LU → HO	α
	3e	2.03	LU → HO-2, HO-3	α
	3f	2.30	LU → HO-5	α
	3g	2.66	LU → HO-11–HO-13	α
